# Supplementary material for: Quantitative analysis of protein interaction network dynamics in yeast
Source: Mol Syst Biol. 2017 Jul 13;13(7):934. doi: 10.15252/msb.20177532 (PMC5527849; doi:10.15252/msb.20177532)
Supplement: Supplementary file 1 — Expanded View Figures PDF [file MSB-13-934-s001.pdf]

Expanded View Figures

Figure EV1. Construction of a genome-scale pool of barcoded PCA strains.

A Haploid strains expressing mDHFR-fragment (F[1,2] or F[3])-tagged proteins of interest were mated and diploids were selected. Interaction between the two proteins was then verified by individually growing each diploid strain in liquid minimal media supplemented with methotrexate (MTX) for 3 days. The F[1,2]-containing haploids (from verified interactions) were then transformed with unique TagModule cassettes, mated with their matching F[3]-containing partner strains, and combined to create a pool (depicted by differently colored yeast cells).

B Growth rate of all 2,394 PCA strains initially constructed and measured as the area under the OD curve (see Materials and Methods) after 75 h of growth in selective media. A cutoff of 8 was applied to identify strains that grew under MTX selection (n = 1,701).

C Relating strain growth [$\log_{10}$(AUC)] to the cellular concentration of the least abundant protein [$\log_{10}$(PPM) – values taken from PaxDB] in each protein–protein interaction pair. Each dot represents one strain, and the line of best fit is shown in red.

D Abundance of proteins involved in previously published PCA complexes (Tarassov *et al*, 2008) that were confirmed (YES) or not confirmed (NO) to grow under the conditions in this study. Protein abundance values were retrieved from PaxDB (Wang *et al*, 2012) and $\log_{10}$-transformed. In the plot, for each strain representing a protein complex, the value of the less abundant protein in that interaction was used. Box boundaries indicate 1st and 3rd quartiles, with the central line indicating median. Bottom and top whiskers extend for either 1.5 times the interquartile range or to the most distal data point, whichever yields the shortest whisker.

E Paired scatterplots comparing raw fluorescence intensities of the six control samples (DMSO) in selective media. Numbers in the lower half indicate the Pearson's r for each pairwise comparison.

F Scatterplot comparing the growth rate of each strain as measured by barcode abundance following competitive growth (x-axis), to that measured in isogenic culture (y-axis). The x-axis represents $\log_2$-transformed ratio of normalized fluorescence following selective and non-selective growth ($\log_2$(+MTX/−MTX)).

 © 2017 The Authors

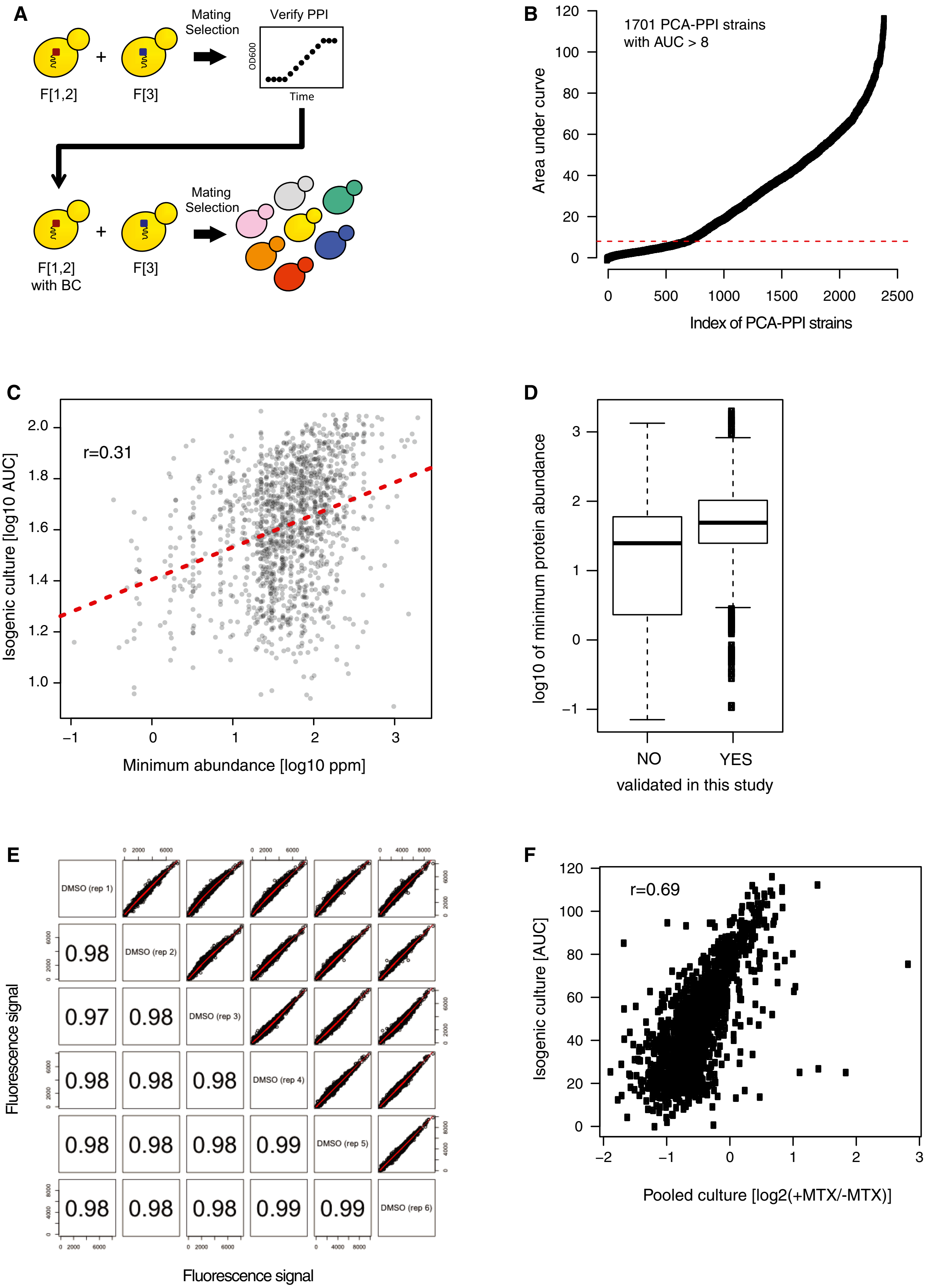
A
Mating
Selection
Verify PPI
OD600
Time
F[1,2]
F[3]
F[1,2]
with BC
B
1701 PCA-PPI strains
with AUC > 8
Area under curve
Index of PCA-PPI strains
C
r=0.31
Isogenic culture [log10 AUC]
Minimum abundance [log10 ppm]
D
log10 of minimum protein abundance
NO
YES
validated in this study
E
DMSO (rep 1)
DMSO (rep 2)
DMSO (rep 3)
DMSO (rep 4)
DMSO (rep 5)
DMSO (rep 6)
0.98
0.97
0.99
Fluorescence signal
F
r=0.69
Isogenic culture [AUC]
Pooled culture [log2(+MTX/-MTX)]

Figure EV1.

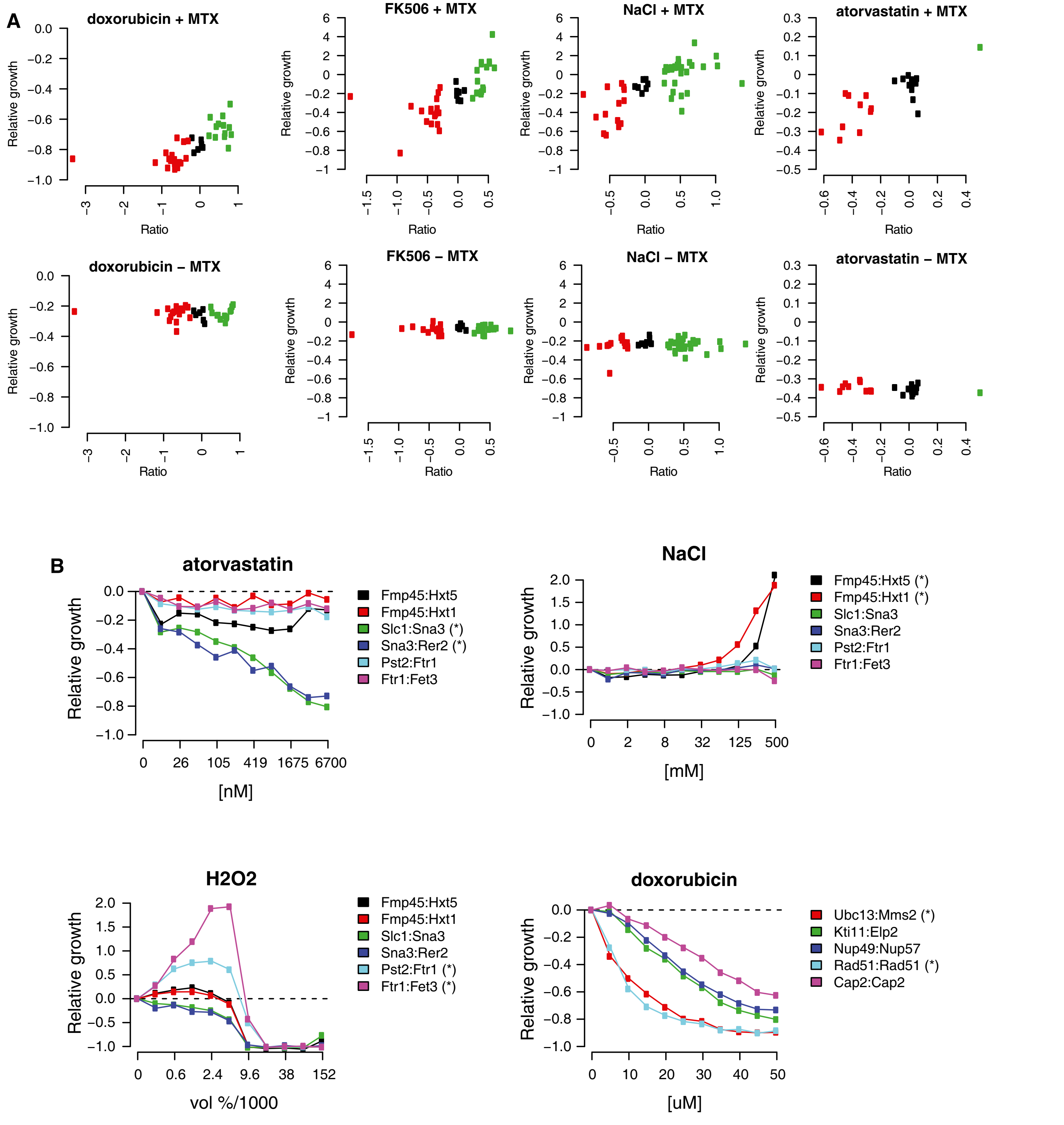

Figure EV2. Confirmation of pool data with isogenic culture.

A Multiple strains that displayed enhanced or diminished growth (green and red, respectively) in the pool screen in doxorubicin, FK506, NaCl, or atorvastatin, as well as several control strains (black), were grown in isogenic cultures. Relative growth values were calculated for either 300 OD measurements (doxorubicin) or 150 OD measurements (FK506, atorvastatin, and NaCl) and compared to ratios derived from the pooled experiment.

B Several PCA strains (indicated in the legend) with enhanced or diminished growth rates identified in the pool screen in NaCl, atorvastatin, hydrogen peroxide, and doxorubicin were grown in selective media in the presence of multiple concentrations of the four agents (indicated on the *x*-axis of each plot). The strains that were identified in the pool assay in each condition are marked with an asterisk. Growth relative to that in the standard environment is indicated on the *y*-axis.

 © 2017 The Authors

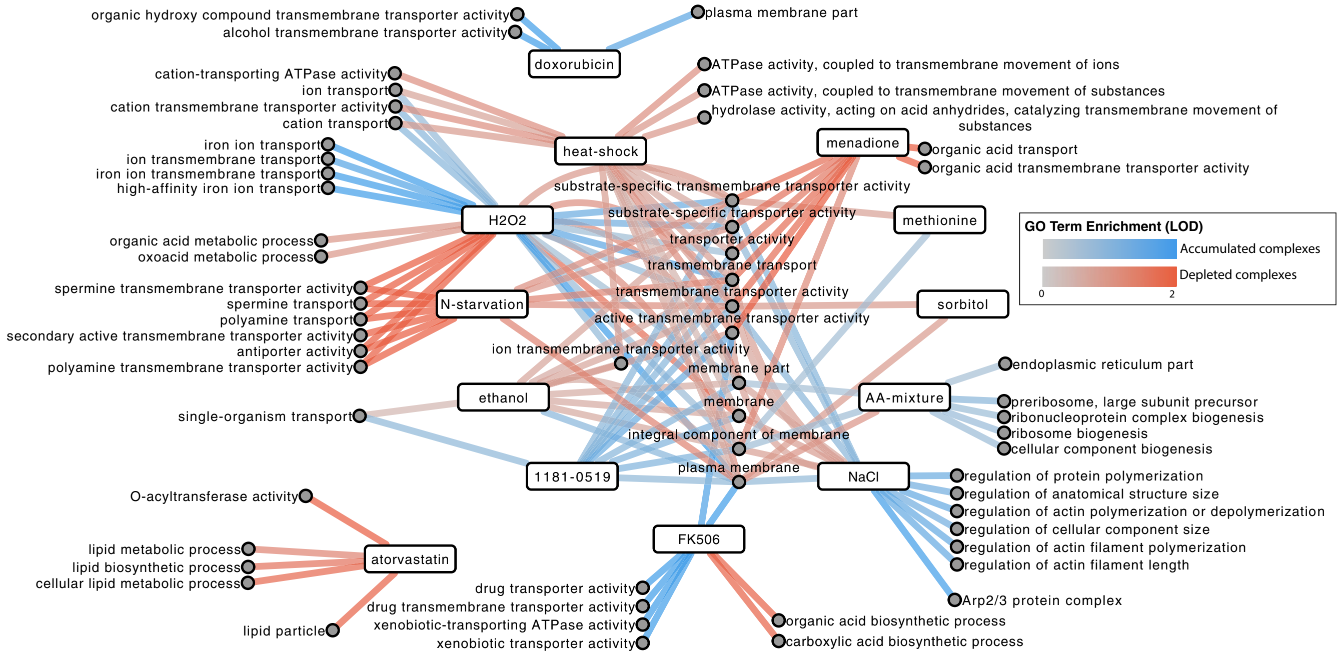

Figure EV3. Expanded GO term enrichment.

Network illustrating functional trends of genes participating in dynamic complexes. For each condition, we assessed functional enrichment among genes participating in either accumulated complexes (blue) or depleted complexes (red). Lines connect terms to their respective condition(s) and are shaded by degree of GO term enrichment measured using the log-odds score (LOD).

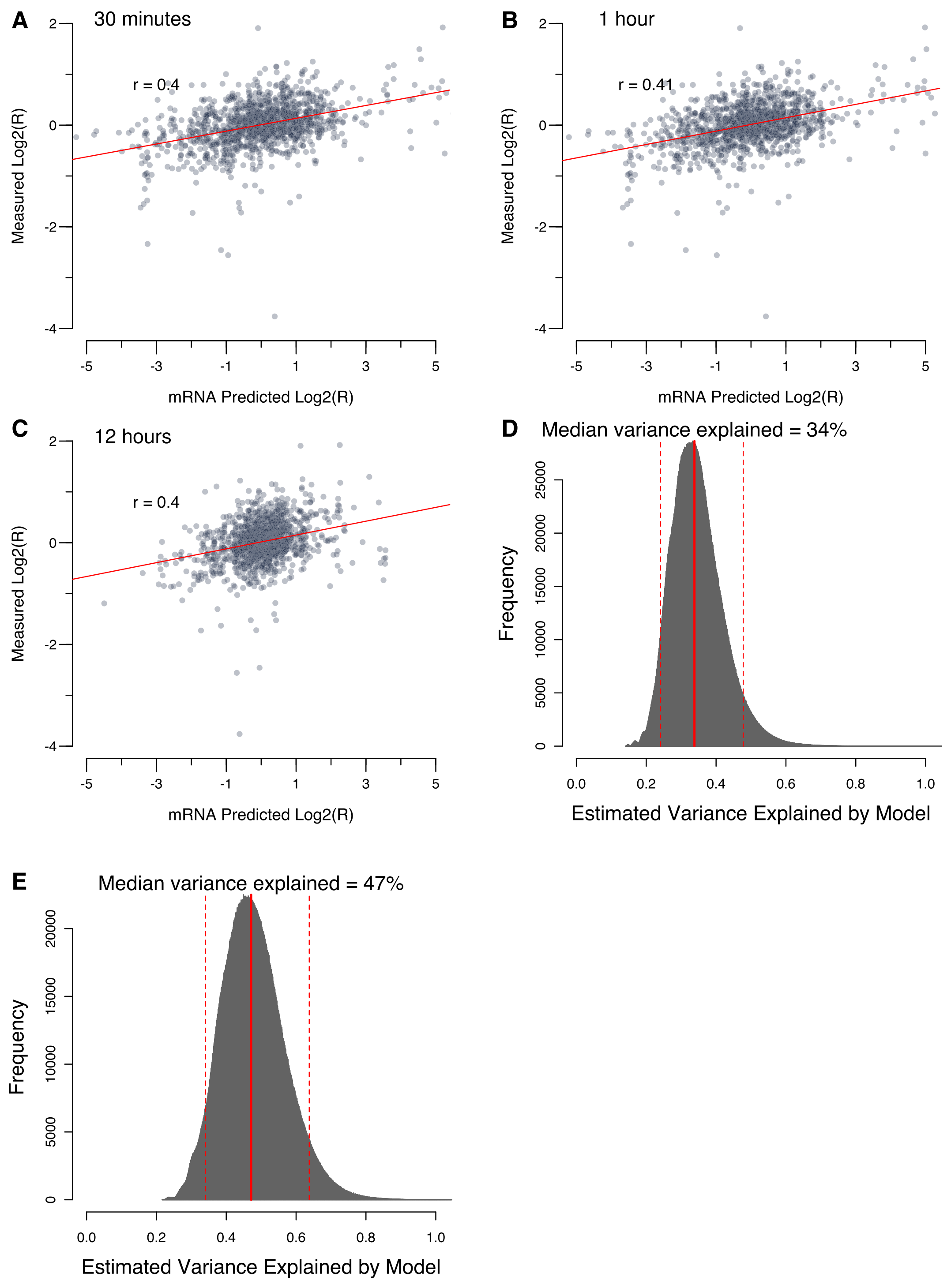

Figure EV4. Accuracy of mRNA-based protein complex predictions.

A–C Protein complex abundance changes measured by BC-PCA compared to mass-action-predicted complex abundance changes based on mRNA expression data under the respiratory growth condition (i.e., in ethanol-containing medium). Each dot represents a protein complex. Predictions were performed using mRNA expression ratios at 0.5 h (A), 1 h (B), and 12 h (C) hours after the shift from glucose to ethanol-containing media.

D, E Distribution of estimates of the proportion of protein complex changes which were explained by mRNA-based mass action predictions for all interactions (D) and interactions found to be significant under ethanol in BC-PCA (E). The red solid line represents the median value, and the red dashed lines represent the 5th and 95th percentile values from 4,950 combinations of "noise-added" randomizations.

 © 2017 The Authors

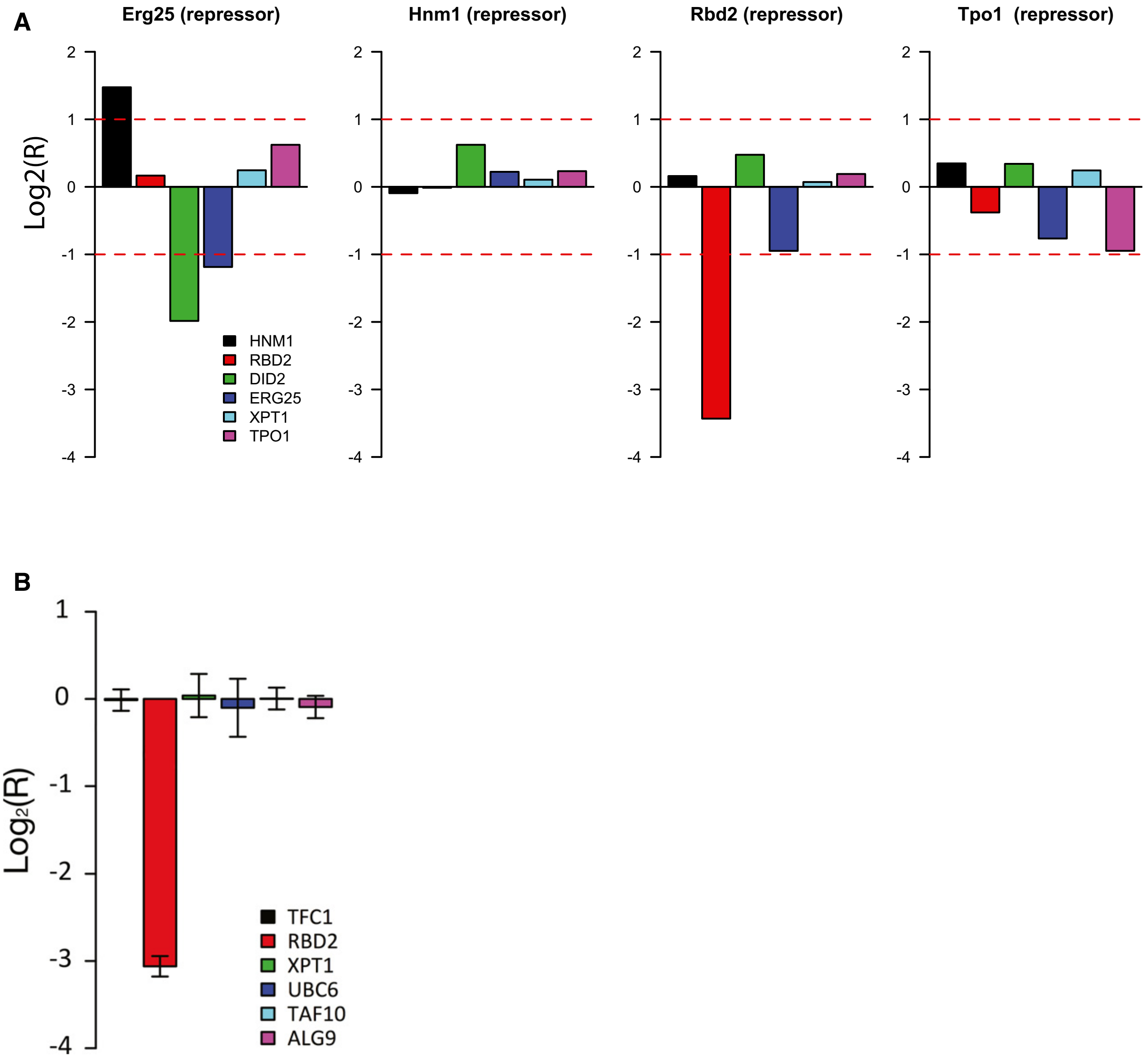

Figure EV5. Knockdown of hub transcripts with CRISPRi. Validation of four CRISPRi guide RNAs using quantitative PCR.

A Barplots showing changes in *HNM1*, *RBD2*, *DID2*, *ERG25*, *XPT1*, and *TPO1* transcript levels following expression of catalytically inactive Cas9 fused to the transcriptional repressor Mxi1 (dCas9-Mxi1), and a guide RNA targeting the *ERG25*, *HNM1*, *RBD2*, *or TPO1* gene locus (as indicated in the title of each plot). Data were normalized using *ACT1* as a control.

B Selective repression of the *RBD2* locus using CRISPRi. Barplot showing changes in *RBD2*, *TFC1*, *XPT1*, *UBC6*, *TAF10*, and *ALG9* transcript levels following expression of catalytically inactive Cas9 fused to the transcriptional repressor Mxi1 (dCas9-Mxi1), and a guide RNA targeting the *RBD2* gene locus. Data were normalized using *ACT1* as a control. The mean of three independent replicates is plotted, and error bars represent the standard deviation.

© 2017 The Authors
